# Benchmarking Sequence-Based Compound–Protein Interaction Prediction through Constructing a Debiased Data Set CDPN

**DOI:** 10.1021/acs.jcim.5c02040

**Published:** 2025-11-20

**Authors:** Yang Hao, Bo Li, Daiyun Huang, Lei Fu, Zhiwei Cao, Xin Liu

**Affiliations:** † Department of Hepatobiliary Surgery, Haikou Affiliated Hospital of Central South University Xiangya School of Medicine, Haikou 570208, P.R. China; ‡ Wisdom Lake Academy of Pharmacy, Xi’an Jiaotong-Liverpool University, 683212 Xi’an Jiaotong-Liverpool University, Suzhou 215123, P.R. China; § Institute of Systems, Molecular and Integrative Biology, University of Liverpool, Liverpool L69 7ZX, U.K.; ∥ School of Life Sciences and Technology, Shanghai Jiao Tong University, Shanghai 200230, P.R. China; ⊥ School of Life Sciences, Fudan University, Shanghai 200092, P.R. China

## Abstract

Accurate prediction of compound–protein interactions (CPIs) is critical for drug discovery, but existing data sets often suffer from biases that hinder model generalization. Here, we first highlighted that over-represented molecular scaffolds and imbalanced label distributions can lead to machine learning shortcuts. While existing debiasing approaches often compromise data set diversity, we present Clustering-based Down-sampling and Putative Negatives (CDPN), a novel protocol for constructing a debiased CPI benchmark. CDPN mitigates biases through compound Cluster-level Down-sampling and generates Putative Negatives from unexplored chemical spaces, ensuring balanced label distributions. Using CDPN, we systematically benchmark deep learning-based CPI models, with a particular focus on protein language models. Although systematic evaluation on PDBbind reveals critical limitations in attention interpretability, thorough ablation studies on the CDPN data set identify superior models such as KPGT-Ankh, which exhibits enhanced generalization and virtual screening performance. The top-performing models from benchmark were also integrated into DeepSEQreen, a no-code web server designed to facilitate community feedback and broader accessibility.

## Graphical abstract

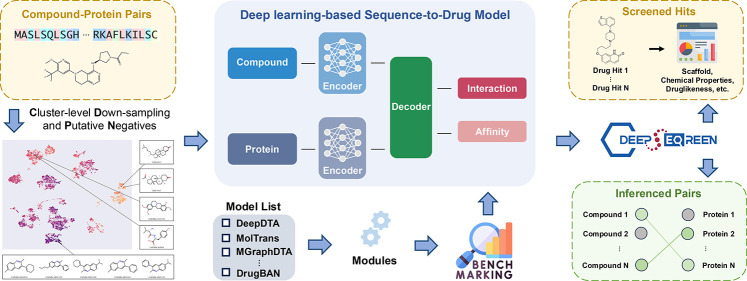

## Introduction

Small-molecule compounds represent over 70% of approved drugs, with their efficacy hinging on specific interactions with protein targets.^1^ Accurate prediction of compound–protein interactions (CPIs) is thus pivotal for identifying hit compounds,^2^ refining the structure of drug candidates,^3^ and forecasting drug interaction profiles to anticipate potential off-target effects^4^ or provide opportunities for drug repositioning.^5^

Existing CPI prediction methods fall into two broad categories: knowledge-based and data-driven methods. Traditional knowledge-based techniques, including molecular docking and molecular dynamics (MD) simulation, can estimate the binding poses and dynamics between compounds and proteins. However, their utility is limited by docking scoring functions and the computational expense of MD simulations, making them inefficient for large-scale chemical space exploration.^6^ In contrast, data-driven methods has the potential to offer promising accuracy and superior scalability for high-throughput screening.

Recently, deep learning-based CPI methods have emerged, designed to learn binding patterns across multiple targets using large-scale, high-quality data sets.^7^ Given that only a fraction of CPIs has experimentally determined 3D structures, these models often use protein sequences as inputs to leverage abundant data while retaining key protein information. Such models are intended to be broadly applicable, facilitating large-scale predictions in both chemical and protein spaces, even in areas with sparse data. To date, over 40 sequence-based CPI methods have been developed, incorporating diverse strategies. Some rely on protein sequence descriptors (e.g., DeepDTI^8^ and DeepAction^9^), while others use embeddings at the amino acid level (e.g., DeepDTA,^10^ GraphDTA,^11^ and DrugBAN^12^). There are also methods that focus on representations of sequence segments (e.g., DeepAffinity,^13^ MolTrans,^14^ and BACPI^15^), and those that integrated predicted structural information (e.g., DrugVQA^16^ and STAMP-DPI^17^). Additionally, certain methods utilize the state-of-the-art protein language models (e.g., AI-Bind,^18^ ConPLex,^19^ and TransformerCPI2.0 ^20^).

Despite years of effort, a fundamental challenge persists: DL models often exploit data set biases by relying on the positive-to-negative ratios of compounds and proteins as shortcuts for prediction, rather than learning the genuine binding mechanisms.^18^ This issue fundamentally stems from two interrelated factors: the shortage of reliable negative samples,^21^ and the under-representation of diverse chemical space in public databases such as DrugBank.^22^ Recent efforts to address data biases have yielded some effective yet limited success. For instance, TransformerCPI2.0 ^20^ attempted to reduce ligand bias by retaining only compounds with both positive and negative labels, yet it failed to resolve imbalanced class label distributions that perpetuate shortcut learning. AI-Bind introduced a network-based negative sampling strategy but was constrained to a narrow chemical space (∼8000 compounds), restricting it utility for large-scale applications. Moreover, expanding CPI training data to large-scale databases like BindingDB and ChEMBL introduces additional complexity, including analogue bias from patent-driven compound clusters and temporal shifts in experimental protocols.^23^

To address these challenges, we introduce CDPN (Clustering-based Down-sampling and Putative Negatives), a novel protocol for curating a debiased CPI benchmark data set building upon available compounds from PubChem. CDPN mitigates analogue bias through cluster-level down-sampling and generates putative negative (PN) samples from unexplored chemical spaces, achieving balanced label distributions at both compound and target levels.

Using CDPN, we systematically benchmarked DL-based CPI models, evaluating the contributions of compound encoders, protein encoders, and interaction decoder modules, with particular emphasis on protein language models (PLMs). Our thorough ablation study on CDPN successfully identified KPGT-Ankh, a novel CPI model that outperforms existing approaches in both benchmark data sets and virtual screening evaluations, demonstrating superior generalization. Additionally, we revealed critical limitations in existing decoder modules, particularly in their interpretability of protein binding sites (systematically evaluated on PDBbind complex structures) and their prediction performance for compound–protein affinity (CPA) and activity cliffs under current data-framework constraints.

The top-performing models from our benchmark, trained on CDPN using a unified PyTorch-based framework, were integrated into DeepSEQreen, a user-friendly web server. We envision this platform bridging the gap between cutting-edge AI methods and drug discovery while facilitating community feedback to further assess the strengths and limitations of existing CPI models. Collectively, this work represents a significant advancement in developing debiased CPI data sets with maximal chemical space coverage, paving the way for more robust and generalizable CPI modeling.

## Results

### Inadequate Generalization of Current Debiased CPI Benchmarking Data Sets

The existing data sets used to construct CPI models vary significantly in scale and coverage, as summarized in Table S1. Davis and KIBA, two widely used kinase data sets, serve as standard benchmarks for most CPI prediction studies. However, they are limited in scale and diversity, covering only 68 and 2111 drugs, respectively. BindingDB and ChEMBL, despite containing millions of interaction records, are often used in their debiased versions, which include only ∼50,000 CPIs^14^ (BindingDB) and ∼250,000 CPIs^20^ (ChEMBL) to mitigate label imbalance.

The debiasing method applied to BindingDB and ChEMBL involves removing compounds with only one type of label (i.e., exclusively active or inactive records against targets, labeled as positive and negative, respectively), preventing models from relying solely on such labels for prediction. However, this trade-off between diversity and label balance (reducing compound clusters from 43,380 to 3729) fails to account for cases where public assay data exhibit extreme label imbalances (e.g., one class being five times more prevalent than another, as shown in Table S2). This oversight creates shortcuts for models to exploit, leading to biased screening results, as demonstrated in our experiments below.

We followed standard procedures to clean the BindingDB and ChEMBL data sets and divided the testing data into four evaluation scenarios (see Methods for details). The data sets before and after applying the “removing one-label-compound” debiasing method are referred to as “Full” and “Reduced”, respectively (Figure 1A). We then trained five representative CPI models on these benchmarked data sets and evaluated their performance (Figure 1B).

**Figure 1:**
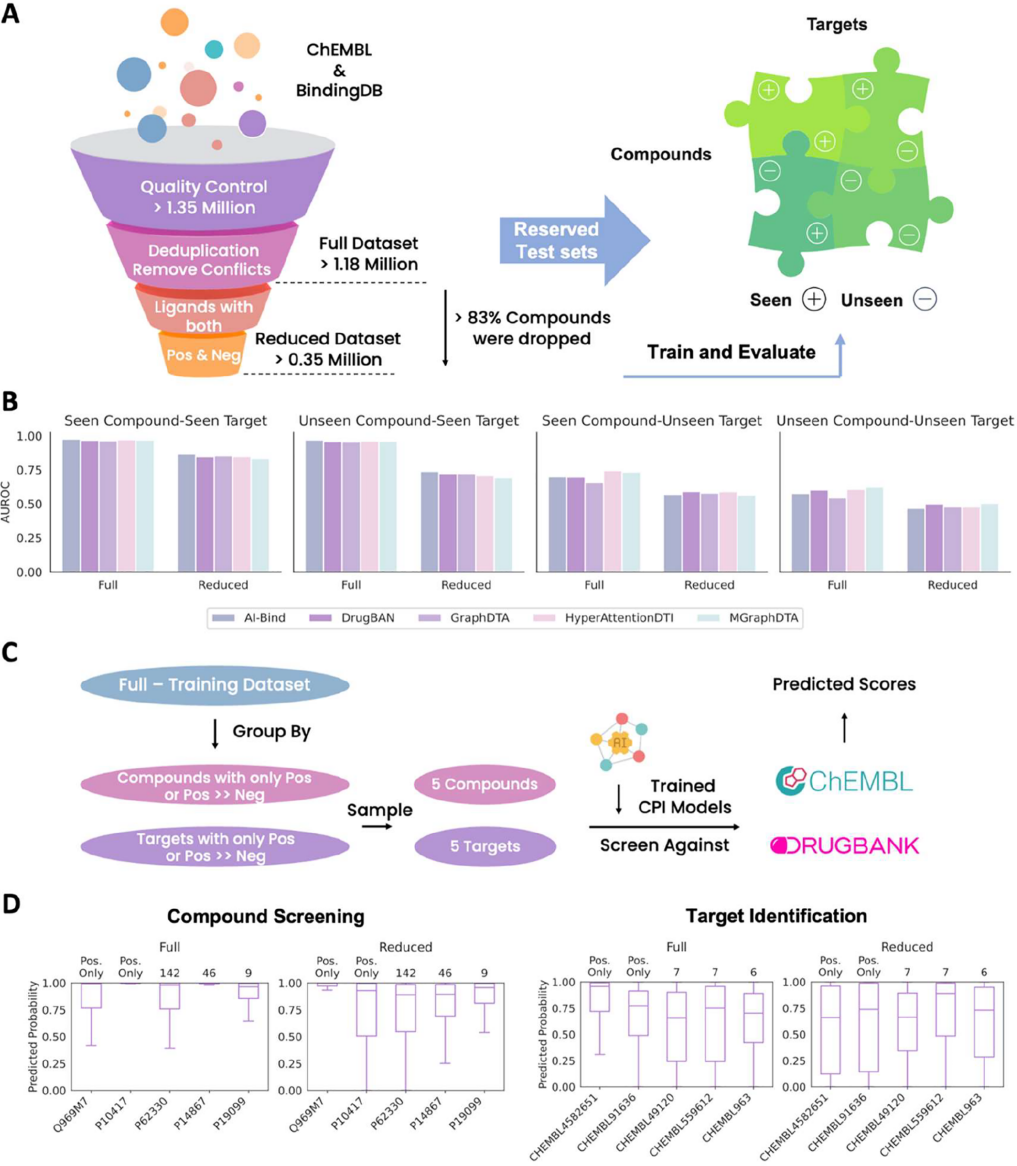
Existing data set construction pipeline and corresponding biased performance. (A) Collection and cleaning large-scale data from previous studies and construction of Full and Reduced data sets. (B) Performance of five representative deep learning-based CPI models on various validation scenarios. The higher the AUROC, the better the performance. (C) Compounds and targets that have disproportionately more positive annotations than negative ones were sampled to conduct virtual screening against targets in ChEMBL and compounds in DrugBank, respectively. (D) Boxplots of the predicted probability score across targets and compounds.

A clear out-of-distribution (OOD) issue emerged. Model performance significantly declined for proteins and/or compounds dissimilar to those in the training set. While the OOD problem has gained increasing attention in recent years, with several studies proposing mitigation strategies,^24^ the inherent biases in data set construction and potential compensatory approaches have received relatively less focus.

To investigate this further, five targets and five compounds were selected with extremely imbalanced label distributions (positive-to-negative ratio ≥ 5:1 or only positive data at present) in training data. We then performed virtual screening against compounds in DrugBank and targets in ChEMBL using the widely adapted baseline GraphDTA^11^ (Figure 1C). The results revealed that models tend to assign disproportionately high probability scores to the majority compounds and targets in the screening library, which contradicts the fact in drug discovery (Figure 1D). This strongly suggests that the model simply memorizes the preference for positive labels among the evaluated targets or compounds, leading it to classify majority of screened molecules as active. Given this bias, it is critical to construct a debiased data set to properly benchmark existing approaches.

### CDPN: A Scalable Protocol for Debiasing Compound–Protein Interaction Data

The most straightforward approach to addressing label imbalance is down-sampling and/or up-sampling existing data to ensure the model learns from a balanced distribution. While this method is widely used in single-target (ligand-based) prediction models, it application to multiple-target (CPI) prediction remains underexplored. Recently, AI-Bind introduced a network-based putative negative sampling method, which selects distal compounds in a CPI network as negatives for given targets. This approach balances label distribution per target and has been shown to effectively prevent models from relying on shortcuts.

However, the chemical space covered by this method remains limited to known active compounds (8111 compounds in AI-Bind study), lacking the structural diversity essential for robust model generalization. Consequently, CPI models trained on AI-Bind data set with network-derived negatives performed poorly when tested on unseen protein targets (see Figure S1). This highlights the remarkable challenge of harmonizing structural diversity with balanced interaction annotations in benchmark data set construction.

To address this, we developed CDPN (Clustering-based Down-sampling and Putative Negatives), a protocol that systematically curates debiased training data sets from large-scale CPI assay data while preserving real-world applicability.

CDPN operates through three synergistic phases. First, chemical space clustering partitions 9.5 million PubChem compounds into 75,745 structurally distinct clusters^25^ using same sampling procedure as MolMap^26^ and k-means clustering with PubChem fingerprints. These clusters expand the known chemical space and ensure representation of both common scaffolds and rare chemotypes (Figure 2A). Second, cluster-aware down-sampling mitigates analogue bias by retaining a maximum of 3 interactions per cluster-label combination (i.e., active or inactive compounds within the same cluster) for each target. This prevents overrepresentation of specific scaffold, reducing bias in model training. Third, putative negative generation balances label distributions at both target and compound levels. For targets with severe positive skewness (positive:negative ≥ 2:1), negatives were sampled from clusters devoid of recorded interactions. For compounds annotated exclusively as positives, negatives were assigned from protein families outside their known targets’ lineages (Figure 2B). The resulting CDPN data set encompasses 3879 protein targets, 289,287 unique compounds, and 741,806 interactions (38.61% positives, 61.39% negatives), spanning five major protein families (kinases: 9.79%, membrane receptor: 8.78%, etc.) with the remaining proteins grouped into “other”. Compared to the Full data set, CDPN reduced scaffold overrepresentation by 37.46%. Detailed settings for CDPN are provided in the Methods section.

**Figure 2:**
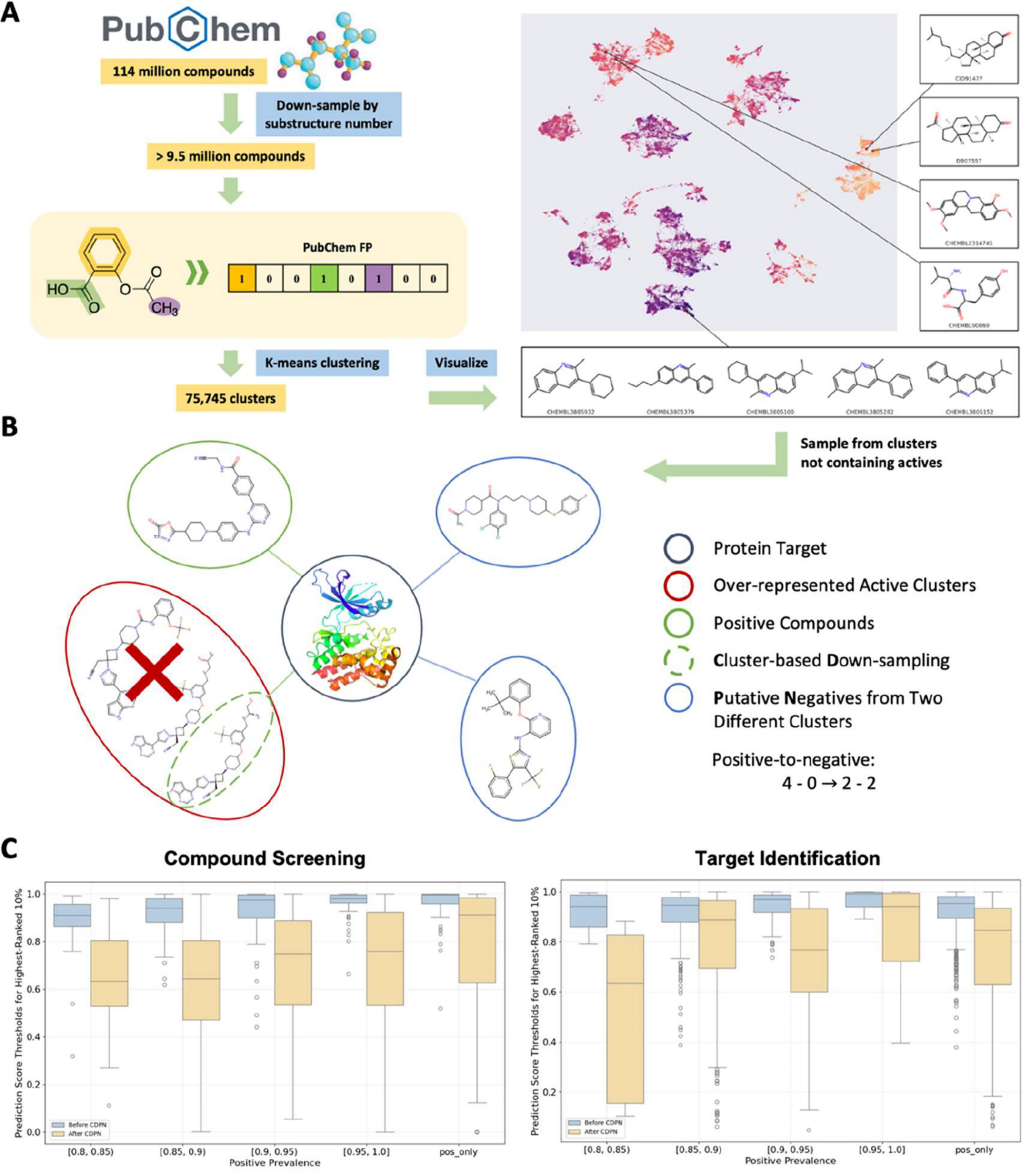
Construct debiased data set using CDPN and the corresponding model performance. (A) Pipeline of the molecule clustering used in this study. (B) Simplified illustration shows one example of clustering-based down-sampling and putative negative sampling. (C) Comparison of prediction score thresholds for the top 10% ranked compounds in virtual screening and proteins in target identification, showing targets and compound clusters with positive prevalence between 0.8 and 1.0 (blue: before CDPN; yellow: after CDPN).

Using the newly developed CDPN protocol, we retrained the classical GraphDTA model on the data set both before and after CDPN processing. We then performed large-scale compound screening against the Enamine library (10,240 diverse compounds) and target identification across 2077 ChEMBL targets, focusing specifically on target and compounds with high positive prevalence, defined as those with >80% positive records in the pre-CDPN data set.

As shown in Figure 2C, prior to CDPN application, the median predicted score for the top 10% of ranked compounds and targets exceeded 0.9, with scores trending upward as positive prevalence approached 100%. In extreme cases, the median score neared 1.0, a clear sign of overconfident and likely biased scoring that renders the screening results unreliable. In contrast, after applying CDPN, the score distribution was substantially lowered and more calibrated, demonstrating CDPN’s effectiveness in mitigating bias and improving prediction reliability.

### Label Distribution and Virtual Screening Generalization Ability Before and After CDPN

To clearly illustrate how CDPN mitigates label distribution bias and thereby enhances model generalization, we first visualized the label distribution across all targets and compound clusters in the data set, both before and after applying the CDPN protocol, using positive prevalence (i.e., the proportion of positive records among all records for a given target or compound, ranging from 0 to 1). Specifically, we employed ESM2 embeddings for proteins and PubChem fingerprints for compounds, followed by UMAP to project these high-dimensional representations into a 2D space for visualization (the closer that points, the more similar the molecules). This approach reveals how label prevalence is distributed across the modeled protein and chemical spaces.

As shown in Figure 3A,B, the pre-CDPN label distribution exhibits a substantial number of targets and compound clusters with positive prevalence close to 1. In contrast, after applying CDPN, most of these highly skewed points shift toward a more balanced positive prevalence around 0.5. Notably, target and compound clusters dominated by negative labels remain unchanged, as we do not introduce putative positive interactions. These inherently negative clusters may reflect genuine biological properties (e.g., targets that are intrinsically difficult to modulate with small molecules) and were therefore retained in the current CDPN implementation. Detailed quantitative information on the data distribution can also be found in Tables S3 and S4.

**Figure 3:**
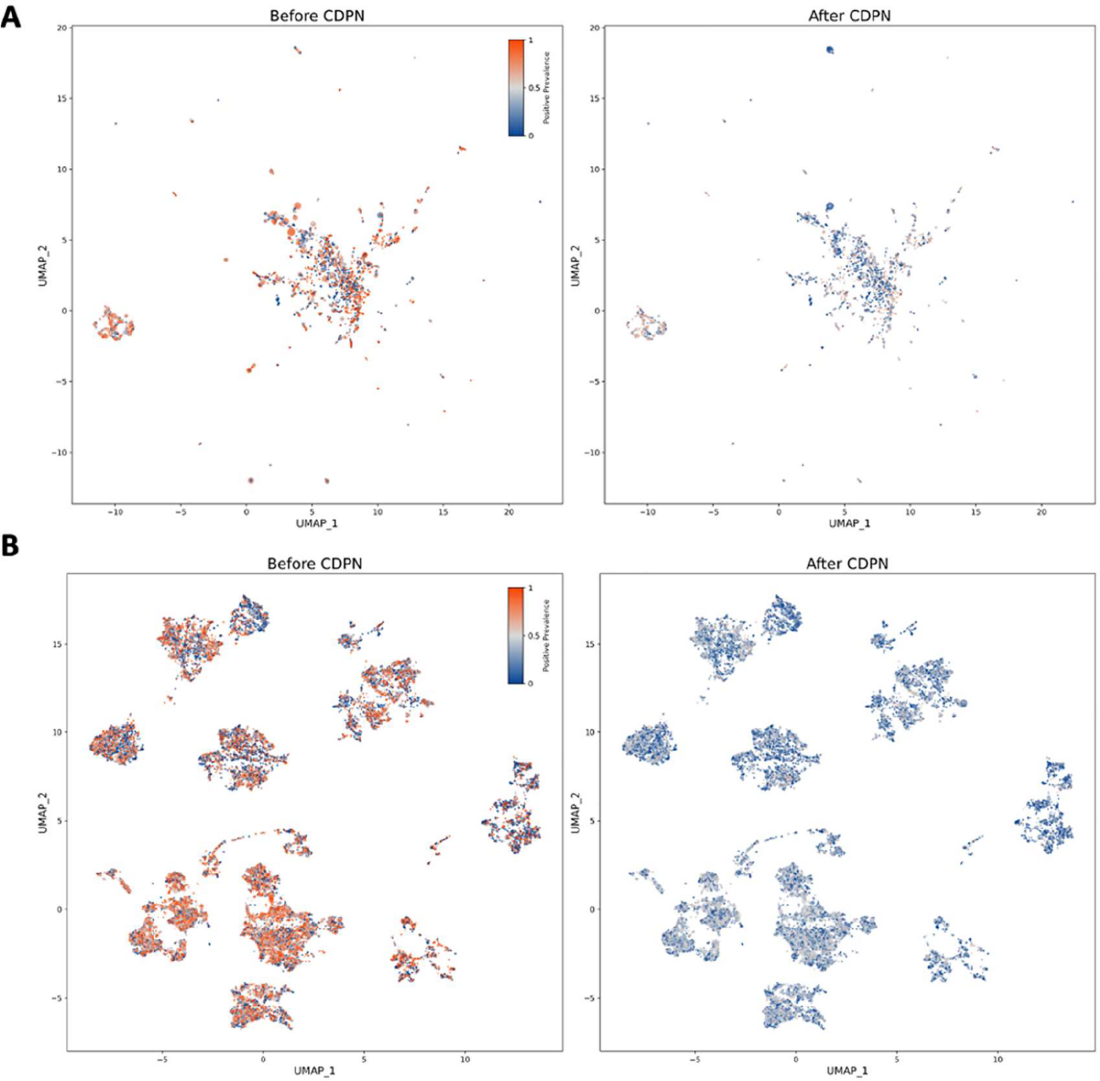
Comparison of label distributions for targets and compound clusters before and after applying the CDPN protocol. (A) UMAP visualization of ESM2 embeddings for all protein targets in the data set before and after CDPN, colored by target-level positive prevalence (red: predominantly positive interactions; white: intermediate prevalence; and blue: predominantly negative interactions). (B) UMAP visualization of PubChem fingerprints for all compound clusters before and after CDPN, colored by cluster-level positive prevalence (color scheme as in A).

To further assess the impact of CDPN on model performance, we trained several established CPI models on both the pre-CDPN and CDPN data sets and evaluated their virtual screening capabilities on the DUD-E (86 seen targets and 16 unseen targets) and Enamine benchmark sets (30 seen targets and 30 unseen targets). As summarized in Table 1, models trained on the CDPN-curated data set outperform those trained on the pre-CDPN data across stringent evaluation metrics BEDROC (α = 80.5) and EF1% for both seen and unseen targets. This demonstrates that CDPN effectively improves the generalization ability of CPI models in virtual screening scenarios.

**Table 1:** Comparison of Virtual Screening Performance Before and After the Introduction of the CDPN Data Set

data set	metric	CDPN	BACPI	DeepConv-DTI	DeepDTA	DrugBAN	GraphDTA	HyperAttentionDTI	MGraphDTA	MolTrans	PMF-CPI	AI-Bind
DUD-E seen target	BEDROC	before	0.11	0.134	0.119	0.294	0.24	0.101	0.147	0.077	0.212	0.029
		after	0.089	0.211	0.114	0.327	0.28	0.145	0.177	0.074	0.278	0.176
	EF1%	before	5.786	7.202	6.466	17.326	14.41	5.175	7.81	4.626	12.085	1.423
		after	4.383	11.78	5.899	18.822	16.149	8.069	9.078	3.997	15.705	11.073
DUD-E unseen target	BEDROC	before	0.042	0.035	0.035	0.026	0.024	0.04	0.081	0.027	0.106	0.045
		after	0.038	0.05	0.039	0.076	0.076	0.06	0.065	0.044	0.131	0.15
	EF1%	before	1.898	1.484	1.737	1.146	0.889	2.001	4.087	1.238	6.069	2.076
		after	1.751	2.622	2.067	3.677	3.932	2.984	3.165	2.145	6.969	9.314
enamine seen target	BEDROC	before	0.206	0.279	0.297	0.516	0.388	0.413	0.26	0.321	0.361	0.07
		after	0.295	0.444	0.346	0.594	0.459	0.479	0.362	0.303	0.545	0.336
	EF1%	before	16.083	22.993	24.78	45.152	33.085	34.388	20.714	26.366	30.804	6.149
		after	25.593	38.529	29.134	52.369	38.957	40.801	29.749	25.321	48.515	28.626
enamine unseen target	BEDROC	before	0.128	0.095	0.151	0.295	0.275	0.191	0.088	0.212	0.17	0.08
		after	0.128	0.173	0.128	0.469	0.317	0.225	0.141	0.197	0.367	0.341
	EF1%	before	11.279	7.814	13.358	28.435	26.012	17.942	7.787	22.121	14.833	7.319
		after	11.385	16.162	11.331	45.948	30.669	21.022	11.343	18.626	34.836	31.75

### Systematic Ablation Study Uncovers Scenario-Specific Strengths in CPI Models

Building upon the CDPN-debiased data set, we then conducted a comprehensive ablation study to dissect the contributions of compound encoders, protein encoders, and interaction modules across diverse drug discovery scenarios. To systematically evaluate their contextual suitability, we selected:

- 13 compound encoders spanning pretrained architectures (KPGT, Mol2Vec), structural fingerprints (ECFP4, RDKitFP, PubChemFP), SMILES-based models (CNN, FCS, LSTM), and graph-based models (GAT, GCN, GIN, MGNN, SAGE);
- 5 protein language models (PLMs), including ProtVec,^27^ TAPE-BERT,^28^ ESM2-8M,^29^ ESM2-15B,^29^ and Ankh Large,^30^ chosen for their parameter diversity and established performance in protein sequence-based tasks;
- 6 Interaction modules comprising three fusion methods (concatenation, Kronecker product, matrix product) and three attention-based methods: bidirectional attention (BIDAT), bilinear two-head attention (BAN), dual-attention gated recurrent network (DAGRN). Fusion methods were included due to their prevalence in prior CPI studies, while attention-based methods were prioritized for their ability to capture local interaction sites, thereby improving interpretability.
- Time-split data set with seen/unseen target and compound cluster scenarios (see Methods for details)

#### Regime-Aware Evaluation of Compound Encoders

The efficacy of compound encoders hinges on their ability to represent structure–activity relationships in a way that generalizes across chemical space. Initial ablation studies, conducted with protein representations fixed to an embedding layer followed by convolutional networks, revealed significant differences in generalization performance (Figure 4A). Pretrained encoders including KPGT (graph-based) and Mol2Vec (SMILES-based), trained on over 10 million molecules to capture hierarchical substructures, demonstrated strong performance in seen cluster–seen target scenario (the chemical cluster and target are present in training set, though never paired), achieving AUROC scores of 0.76 and 0.74, respectively. This suggests they effectively capture target preferences within known chemical clusters, reflecting robust compound representation.

**Figure 4:**
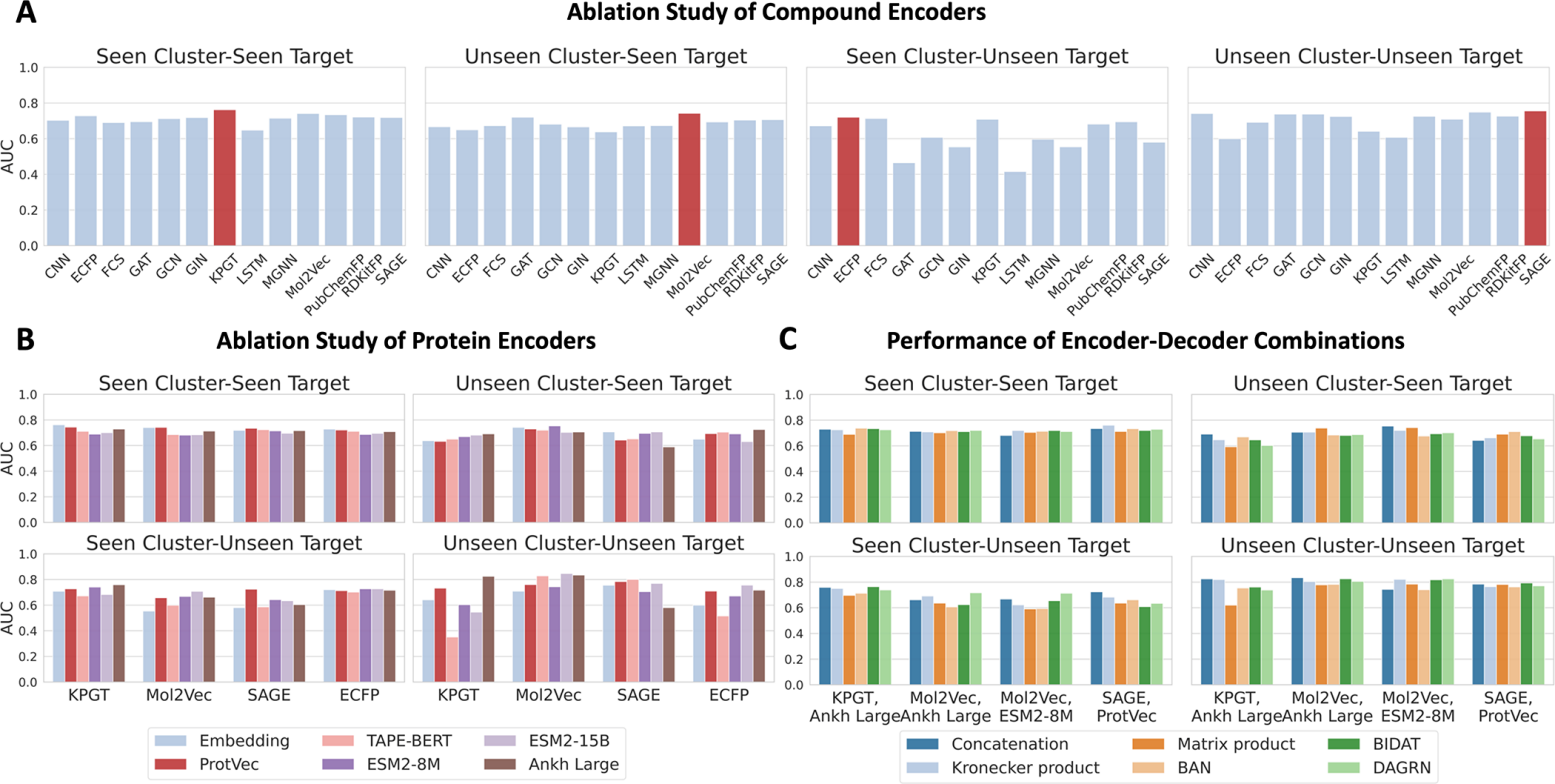
Ablation study of components in CPI model. (A) Ablation study of 13 compound encoders paired with an embedding-based protein encoder. (B) Evaluation of 5 protein language models (with embedding as baseline), spanning a wide range of scheme and parameter sizes. (C) Performance of top-performing encoder combinations integrated with different interaction decoder modules. AUC: AUROC (for scenarios with positive-to-negative ratio >1) and AUPRC (for ratio <1), accounting for class imbalance across the four scenarios (ratios: 1.76, 1.63, 0.52, and 3.24).

However, their generalization capabilities diverged: KPGT performed well in seen cluster–unseen target cases, while Mol2Vec showed promise in unseen cluster scenarios. Among other encoders, topological molecular fingerprints (ECFP) excelled for seen cluster but struggled with unseen cluster. In contrast, GNN-based methods (e.g., GAT and SAGE) performed strongly in unseen cluster scenarios, with GAT ranking second in unseen cluster–seen target and SAGE ranking first in unseen cluster–unseen target. SMILES-based models were less competitive, with only SMILES + CNN showing some efficacy in unseen target cases.

Notably, these conclusions are based on a fixed protein encoder (an embedding layer trained from scratch), whereas CPI prediction inherently involves interactive learning between compounds and proteins. Guided by these findings, we selected KPGT, Mol2Vec, SAGE, and ECFP for subsequent protein encoder ablation studies, ensuring a representative yet tractable evaluation of chemical representation diversity.

#### Regime-Aware Evaluation of Protein Encoders

Recent CPI models have demonstrated that incorporating PLMs as the protein encoders can significantly enhance performance.^20,31,32^ However, the efficacy of different PLMs has not been rigorously benchmarked, and reported performance has been limited to data set constrained by coverage and label bias. To address this, we selected 5 representative PLMs, ranging from the early word2vec-based ProtVec,^27^ to the first transformer-based PLM TAPE-BERT,^28^ to the widely used ESM series (with two versions differing by over 1000 times in parameter size: the smallest ESM2-8M^29^ and the largest ESM2-15B^29^), and the state-of-the-art Ankh Large^30^ model, to evaluate their effectiveness in CPI predictions.

To date, no existing CPI model has fine-tuned PLMs during training. Our preliminary experiments show that both full and parameter-efficient fine-tuning of the lightweight ESM2-8 M can significantly improve performance in unseen scenarios (see Figure S2 for details). However, determining the most effective fine-tuning strategy for PLMs in CPI prediction requires dedicated investigation. In this benchmark, we focus solely on using PLM embeddings as input to CPI models. We evaluate three feature extraction methods on top of these embeddings: average pooling to sequence-level features followed by an MLP, CNN-based processing, and transformer-based processing of amino acid-level features. Our experiments reveal that the more complex transformer architecture does not yield significant performance gains, while CNNs generally outperform MLPs with acceptable computational overhead (see Figure S3 for details).

As shown in Figure 4B, incorporating PLMs did not significantly improve model performance in the both seen scenario. Only ProtVec, one of the earliest PLMs that maps protein sequences into an *n*-dimensional vector encoding biophysical and biochemical properties, performed comparably or slightly better than the baseline embedding (used in the Compound Encoder ablation). This suggests that summarized feature vectors may facilitate easier learning of known data preferences.

A general trend emerged where PLMs enhanced model performance when generalizing to at least one unseen scenario. Advanced PLMs typically outperformed earlier ones when paired with pretrained compound encoders (KPGT, Mol2Vec) or the simple yet effective fingerprint ECFP, but not with the graph-based model SAGE, which benefited most from ProtVec in most cases. While larger language models generally capture richer representations, this advantage did not strictly translate to CPI model. For instance, the largest model, ESM2-15B, did not consistently surpass smaller ones like ESM2-8M and Ankh Large (1.15B). Notably, in the unseen cluster–seen target scenario, ESM2-8M paired with Mol2Vec achieved the top performance. More strikingly, in unseen target scenarios, Ankh Large significantly boosted KPGT-based models, securing the top performance on seen cluster and ranking fourth on unseen cluster, while also delivering 5.1 and 18.4% performance gains over the baseline Embedding for seen and unseen clusters, respectively.

Regarding compound encoders, advanced PLMs reinforced previous findings: KPGT and Mol2Vec retained slight advantages in seen cluster–seen target and unseen cluster–seen target scenarios, respectively. For unseen target scenarios, these two pretrained encoders, aided by PLMs, outperformed ECFP and SAGE. Notably, neural network-based compound encoders only significantly surpassed ECFP in both unseen scenarios, highlighting both the generalization power of neural representations and the need for more advanced methods.

Since the interaction module (which merges compound and protein representations for final prediction) could also influence performance, we selected the aforementioned combinations (SAGE-ProtVec, KPGT-Ankh, Mol2Vec-ESM2-8M) as well as Mol2Vec-Ankh (given the individual strengths of Mol2Vec and Ankh) for the final ablation study.

#### Regime-Aware of Interaction Decoders

Surprisingly, the attention mechanism, designed to guide the model toward key atoms and residues involved in CPI, did not yield significant performance improvements in our benchmark.

A distinct improvement was only observed for Mol2Vec-based models in unseen target scenarios. Among the best combinations for each scenario, only in the seen cluster–unseen target case did bidirectional attention (BIDAT) provide a marginal 0.5% performance gain oversimple concatenation (the baseline) for the KPGT-Ankh combination.

In other scenarios, attention mechanisms provided no clear benefits. The SAGE-ProtVec-Kronecker combination (first place) surpassed KPGT-Ankh-BAN (second place) by 2.2% AUC in both seen scenario. Meanwhile, Mol2Vec-ESM2-8M with standard fusion maintained top performance for unseen cluster–seen target predictions. For both unseen scenario, Mol2Vec-Ankh-Concatenation delivered the best results, followed closely (0.9% lower) by both Mol2Vec-Ankh-BIDAT and KPGT-Ankh-Concatenation in second place. These suggest that the attention-based interaction module, without direct supervision, provide no distinct advantage in predictive performance.

This finding prompted us to investigate another critical aspect of attention mechanisms: interpretability. Specifically, we examined whether these modules accurately identify sequence regions forming druggable binding pockets or residues involved in key interactions (e.g., hydrogen bonds). While prior studies have reported success in specific cases, a systematic large-scale evaluation of this capability remains lacking. To address this gap, we leveraged protein–ligand complex structures from PDBbind 2020 for assessment.

After data cleaning, 9868 complexes with annotated pocket residues were analyzed (Figure 5A). Ideally, residues assigned high attention weights should correspond to pocket regions. However, despite varying coverage across interaction frameworks, the interaction-overunion (IoU) scores remained consistently low (Figure 5B,C), indicating that models frequently assigned high weights to nonpocket regions. A similar trend emerged when using PLIP-identified interaction residues as ground truth (Table S5).

**Figure 5:**
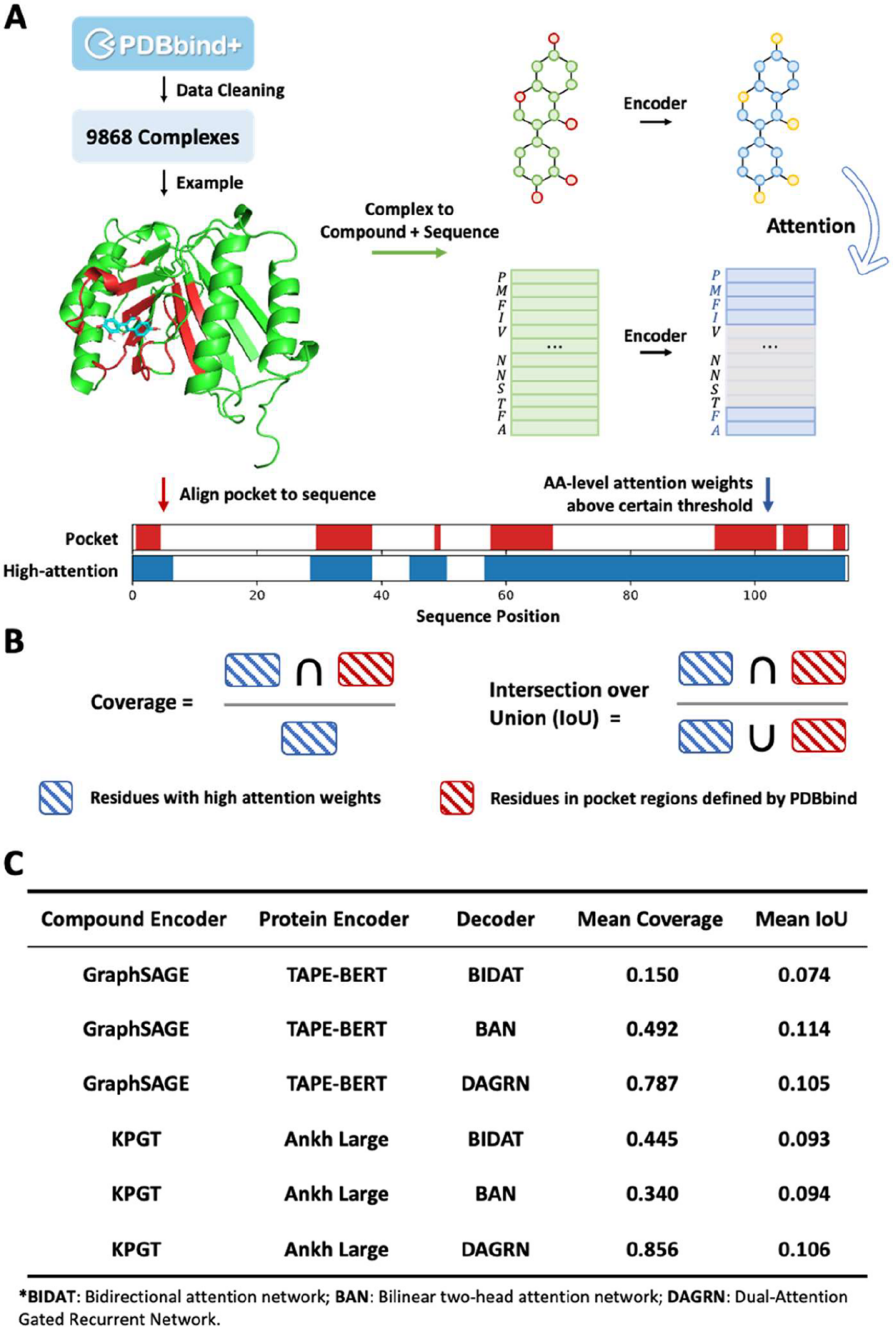
Systematically evaluation of interpretability of attention modules on PDBbind. (A) Simplified pipeline for evaluation. (B) Definition of two evaluation metrics. (C) Performance of six combinations. GraphSAGE-TAPE and KPGT+Ankh combinations were selected due to their promising performance on both unseen scenarios.

While this does not necessarily negate the model’s interpretability, as the model may instead rely on other domains to identify protein family specific features, it highlights a critical limitation. Future work could explore integrating structure-aligned PLMs or incorporating known binding pocket information as an additional supervisory signal for the attention module.

### CDPN Enhances the Development of Novel Effective CPI Models

Despite ongoing challenges, our newly developed CDPN data set facilitates the development of novel effective CPI models with promising virtual screening performance. This was demonstrated through virtual screening evaluations using an Enamine library of 10,240 diverse compounds (covering 7253 distinct clusters), along with 20 randomly selected seen targets and 10 unseen targets. For comparison, we selected three high-performing baseline models, GraphDTA-GIN, MGraphDTA, and DrugBAN, due to their strong performance in both seen, seen cluster—unseen target, and unseen cluster scenarios, respectively. For models from ablation study, we included KPGT-Ankh-Concatenation/BIDAT and Mol2Vec-ESM2-8M-Concatenation.

Figure 6A,B reveals that while all models exhibit comparable virtual screening performance on seen targets (consistent with benchmark results), our ablation models demonstrate significant advantages for unseen targets. The KPGT-Ankh-Concatenation model outperformed DrugBAN by 3.6% in AUC on benchmark tests and showed a 7.8% improvement in median EF1% during virtual screening evaluation. To translate these benchmark results into practical drug discovery applications, we conducted case studies using two challenging targets (unseen cluster-unseen target scenario): the orphan receptor ADGRD1 (Adhesion G-protein coupled receptor D1) and PSEN2 (Presenilin-2), both having only one known drug in DrugBank.

**Figure 6:**
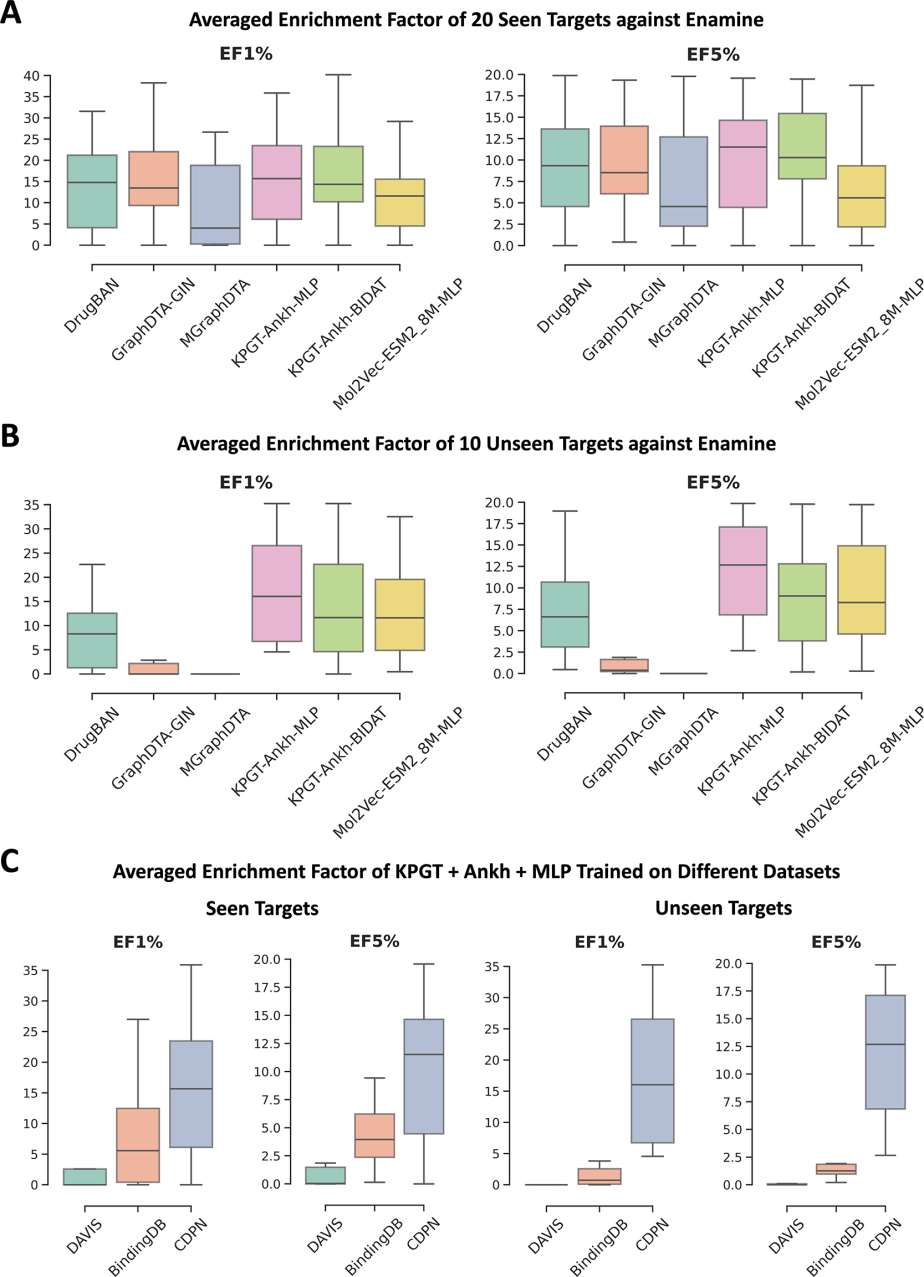
Comparison of new combinations and existing models on virtual screening. (A) Virtual screening performance on 20 seen targets. (B) Virtual screening performance on 10 unseen targets. (C) Comparison virtual screening performance of same model trained on different data sets.

Tables S6 and S7 demonstrates the superior virtual screening capability of our approach. For ADGRD1, KPGT-Ankh combinations successfully ranked the known drug *Onapristone* at positions 27 (Concatenation) and 92 (BIDAT), while existing models placed it between 3787 and 4700. While DrugBAN and GraphDTA performed well for PSEN2 (ranking the known drug at 13th and 111th positions), our KPGT-Ankh combinations maintained competitive performance (25th and 56th positions).

To further validate our approach, we retrained the KPGT-Ankh model on DAVIS and BindingDB data sets, which are commonly used for developing existing CPI models. As shown in Figure 6C, models trained on the CDPN data set consistently outperformed those trained on classical data sets in virtual screening tasks. These results collectively highlight CDPN’s potential to enhance both the performance and generalizability of CPI models, offering a robust framework for developing more accurate and reliable drug discovery applications.

### Deepseqreen: An Integrated Sequence-Based Deep Learning Webserver for Virtual Drug Discovery

After systematically evaluating deep learning-based CPI models and addressing potential biases, we integrated the top-performing models into a freely accessible Web server named DeepSEQreen. DeepSEQreen provides a comprehensive suite of sequence-based CPI models for drug hit screening and interaction pair inference Figure 7. Based on user input and our benchmark results, the platform automatically provides model recommendations. Beyond interaction probabilities, DeepSEQreen also calculates essential chemical properties to facilitate user-friendly hit candidate filtering. Designed for accessibility, DeepSEQreen enables users without programming expertise to seamlessly leverage advanced AI techniques for drug discovery. By bridging the gap between cutting-edge AI and practical drug development, DeepSEQreen also serves as a platform for community feedback, helping identify strengths and limitations in existing sequence-based CPI models. DeepSEQreen is freely available at: https://www.ciddr-lab.ac.cn/deepseqreen/.

**Figure 7:**
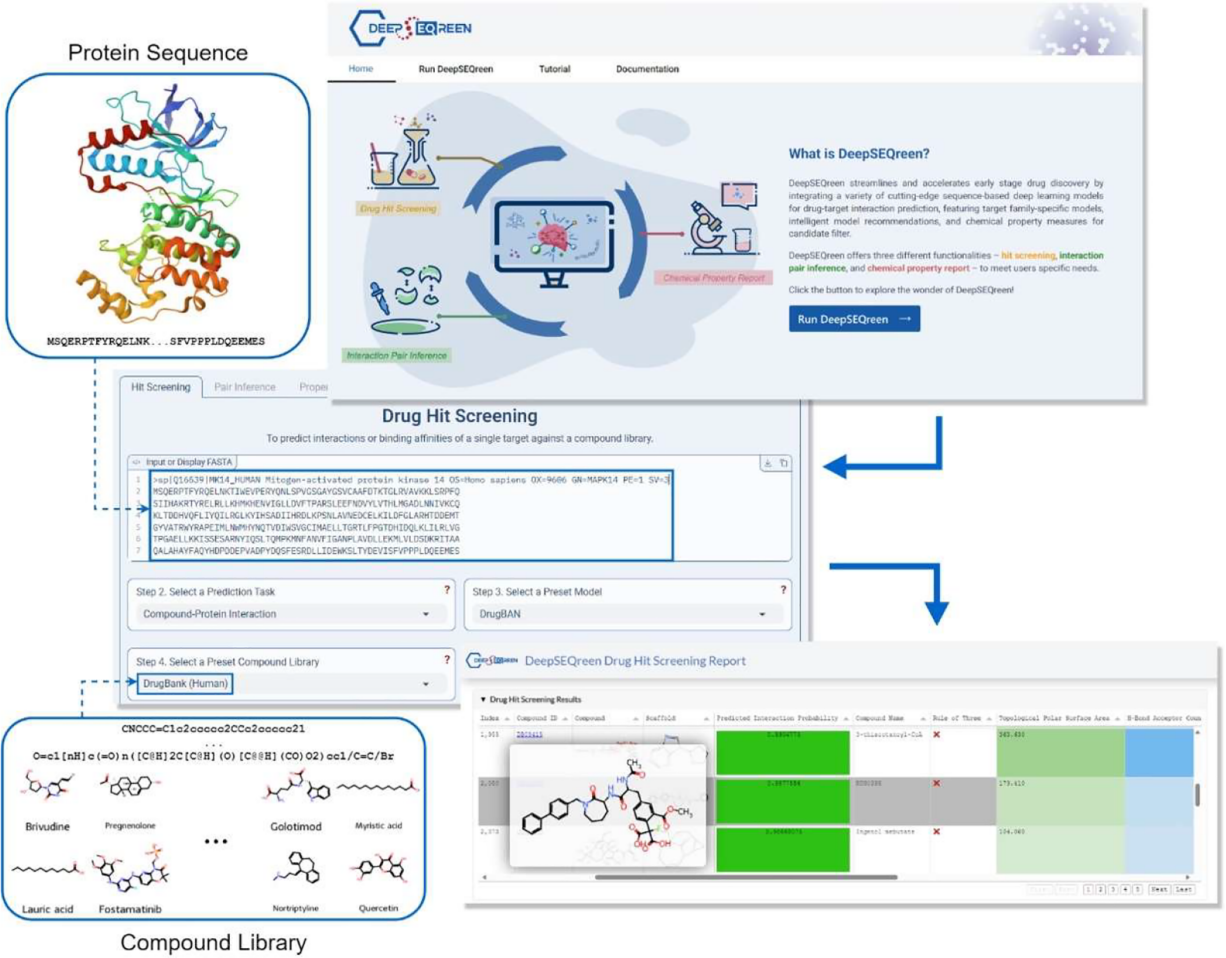
A simplified illustration of drug hit screening task in DeepSEQreen. Users only need to input the sequence of the target of interest, select a preset model or let the DeepSEQreen recommend the model based on our benchmark results, and then choose a preset compound library or upload their own library. After selecting the library, they can click “Run” to start the prediction. Once the task is completed, a report with the predicted interaction probabilities and the calculated properties of the selected compounds will be available for online viewing or download.

## Discussion

Unlike ligand-based virtual screening models, which focus on a single entity, CPI prediction involves two entities, introducing significantly greater complexity in addressing out-of-distribution challenges. The scale of existing data remains too limited to adequately cover the joint distribution space. Moreover, compounds tested for different targets are often dissimilar, resulting in a sparse and fragmented observed distribution. Given the varying data sizes per target and imbalanced label distributions, we hypothesize and provide evidence that models may exploit label imbalance as a shortcut, memorizing positive-to-negative ratios for trained entities rather than genuinely learning the underlying joint distribution. To address this, we propose CDPN, envisioning it as a foundational step toward a high-coverage, debiased data set for developing robust CPI models.

After demonstrating CDPN’s effectiveness, we re-evaluate existing models through ablation studies to identify modules critical for CPI prediction. In light of recent advances in PLMs, we systematically assess their utility in CPI tasks. Our analysis reveals that pretrained compound and protein encoders enhance generalization under certain scheme. For instance, when using raw PLM representations as input, we find that complex transformer architectures offer no clear advantage oversimpler CNN frameworks. Model performance depends more on training schemes than parameter size. Ankh Large (1.15B parameters), despite being 10× smaller than ESM2-15B, matches or surpasses the latter in certain scenarios, likely due to its protein-specific optimization. Preliminary experiments also suggest that fine-tuning PLMs within CPI models can improve generalization, pointing to efficient fine-tuning strategies as a key direction for future work.

However, challenges remain. While CDPN effectively incorporates putative negatives to balance label distributions for the majority of proteins and compounds, it cannot address targets or compound clusters that exhibit extremely high negative prevalence, since we cannot reasonably assume any putative positives without biochemical evidence. Such skewed distributions may intrinsically reflect the target druggability or compound inactivity. From a data science perspective, however, these imbalances can still introduce bias into model training.

Furthermore, sequence-based CPI models aim to learn sequence–structure–binding mappings, but limited data hampers progress, as evidenced by poor performance on PDBbind (attention vs pocket). As community-wide data accumulation will take time, integrating existing protein pocket knowledge may advance both performance and interpretability. Promising directions include fine-tuning sequence-only models on structure-based data sets like PDBbind using binding pockets as supervision (e.g., via pocket residue prediction or attention-guided losses). Moreover, newer protein language models increasingly align sequence with structure during pretraining, offering more structure-aware representations for CPI tasks even without explicit 3D inputs.

Beyond CPI prediction, we benchmarked our model on compound–protein affinity (CPA) prediction. However, regression tasks are far more challenging than classification, requiring larger data sets to model smooth joint distributions while remaining difficult to balance, further complicated by unresolved issues like activity cliffs, even in ligand-based tasks. Despite efforts to adapt CDPN for affinity scenarios, including various strategies to assign low-affinity values to putative negatives (Figures S4 and S5), CPA benchmarks show that CDPN yields at most modest improvements, limited to certain unseen target scenarios. This highlights the need for regression-balanced data sets to advance sequence-based CPA models.

In conclusion, we present CDPN, a data set construction pipeline that mitigates label imbalance bias while preserving chemical space coverage. Through ablation studies, we guide encoder selection for CPI models and highlight the promise of combining tailored PLMs with compound encoders. We also identify limitations in current decoders, positioning this work as a foundation for future benchmark data sets and CPI model development.

## Methods

### Data Preprocess

To ensure timeliness of the models, we harvested latest activity data from several databases, namely ChEMBL v33, BindingDB v202312, and PDBbind v2020. The data sets underwent extensive cleaning and integration processes suitable for training and diverse evaluations.

For ChEMBL, our data processing procedure, aimed at preserving high-quality data, is detailed below:

a) Assay selection: only assays with the highest confidence score and assay type “binding” or “functional” were retained.
b) Source consideration: out of the 65 sources available in ChEMBL, we focused on four major ones that cover the majority of high-confidence assays: scientific literature, patents, and data exchanged from other reputable databases including PubChem BioAssay and BindingDB.
c) Activity unit filter: only records with activity units in (Potency, *K*_i_, *K*_d_, IC50, AC50, EC50, ED50, XC50) were retained. These units align with the definition of pChEMBL value, allowing for a meaningful comparison of measures related to half-maximal response concentration, potency, or affinity on a negative logarithmic scale.
d) Data validity check: data with validity flags indicating values outside the typical range for that activity type, or potentially erroneous data, were removed.
e) Drug-likeness filters: compounds without metal atoms, with a molecular weight less than 1000 Da, and a number of heavy atoms greater than 12 were retained for modeling. This step aimed to eliminate small or inorganic compounds that are not representative for modeling the chemical space pertinent to normal drug discovery projects. While this rule is more lenient than the Lipinski rule-of-five, the objective was to retain as much valuable chemical information as possible while still excluding nondrug-like compounds.
f) Frequent hitter filters: colloidal aggregators^33^ and luciferase inhibitors^34^ are two known contributors to frequent hitters^35^—compounds that can form nonspecific bonds with various macromolecular targets or interfere with elements of the assay formats or techniques, leading to false positives. We collected known aggregators from Aggregator Advisor and known luciferase inhibitors from 42 PubChem Bioassays. For compounds showing positive effects in ChEMBL data but also recognized as potential aggregators or luciferase inhibitors, we removed them from the training data to avoid potential false positive labels.

For BindingDB, we downloaded all data stored in the version dated 202312. Data sourced from ChEMBL were removed since we had already processed the ChEMBL data separately. For the remaining data, only records involving single proteins and small molecules with dose–response values were retained. These dose–response values were measured by at least one of the following metrics: EC50, IC50, *K*_d_, or *K*_i_, and thus all were retained. The same drug-likeness and frequent hitter filters applied to the ChEMBL data were also applied to the BindingDB data set.

After data cleaning, we merged data from ChEMBL, BindingDB, and PDBbind. A threshold of 1000 nM (pChEMBL = 6) was selected to divide the data into active (positive) pairs and inactive (negative) pairs. Records with conflicting labels from different sources were removed. The processed data set (named as Full data set) was then randomly split for initial benchmarking, construction of Reduced data set, and construction of CDPN data set.

The construction of CDPN relies heavily on the robust clustering of compounds. Following recent works that group compounds into families and successfully apply them to ligand-based deep learning, we adopted a similar approach. First, similar to MolMap,^26^ we downloaded 114 million molecule entries from PubChem and deduplicated them based on their canonical SMILES, resulting in 110 million unique molecules. These unique molecules were then grouped into 100 classes according to the number of bits in their ECFP4 molecular fingerprints, calculated using RDKit. In ECFP4, a higher number of bits indicates a greater substructure complexity. This allowed us to sample compounds while retaining the distribution of substructure complexity. In total, 9.5 million compounds were sampled from these 100 classes to reduce computational costs while maintaining molecular diversity.

For clustering, we chose the PubChem fingerprints, which are used by PubChem for similarity neighboring and searching. Dimensionality reduction was performed on the 881-bit fingerprints, selecting enough components to retain 95% of the total variance. After dimensionality reduction, we systematically evaluated a range of K values using distance-based metrics (total within-cluster sum of squares and RMSE), but no distinct inflection point was observed (Figure S6). Given these observations and Xiao et al.’s^25^ large-scale assessment, we adopted *K* = 75,745, which still provides broad coverage of the experimentally explored druggable chemical space (Figure S7). The K-means clustering was performed using the Faiss library (https://github.com/facebookresearch/faiss), which supports fast and robust clustering even at the scale of 9.5 million compounds.

### Representative Down-Sampling and Data Augmentation Using Putative Negatives

#### Down-Sampling

Specifically, we aim to retain compounds with dose–response data for multiple targets as much as possible to reduce data sparsity. Therefore, we first selected records relevant to multitarget compounds for down-sampling. At most three records from each unique pair of cluster and target combination were retained. For the remaining training data set, we down-sampled to retain at most three records from each unique combination of target, compound cluster, and label class. The two subsets were then joined to perform up-sampling.

#### Putative Negative Compounds for Targets

Since putative positives cannot be determined without experimental validation, we only up-sampled with PN compounds for targets that have only positive records or where positives significantly outnumber negatives to achieve target-level balance. Specifically, we used a rectified half sigmoid function (see equation bellow), which maps the original target-level negative prevalence to a target postsampling ratio. Targets with negative prevalence ≥0.5 remain unchanged, whereas under-represented targets (<0.5) are smoothly upweighted. The steepness of the scaling is controlled by the parameters *g* = 3 and m = 0.4, chosen empirically to correct bias without introducing spurious noise (Table S8).

$$\alpha_{T_{i}}=(x,g=3,m=0.4)=\max\left(x,\frac{2-2m}{1+e^{-g\cdot (x-1)}}+m\right)$$ 1

$$N_{T_{i}}^{\mathrm{PN}}=\alpha_{T_{i}}\cdot N_{T_{i}}^{P}-N_{T_{i}}^{N}$$ 2

To reduce sparsity, for any given target, we first used compound clusters with only positive interactions and no recorded interaction with the target as the source of PN compounds. With no more than three compounds per cluster, we then perform random representative sampling of PN compounds by cluster to obtain the required number of putative negatives, ensuring cluster diversity, up to the defined objective number. The effect of this procedure on the target-level negative prevalence is illustrated in Figure S8.

#### Putative Negative Targets for Compounds

We also up-sampled with PN targets to achieve compound-level balance. For compounds, we relied on clusters to measure compound similarity. For proteins, we grouped them into six families: Kinase, Nonkinase Enzyme, Nuclear Receptor, Membrane Receptor, Ion Channel, and Others. We assume that a random compound cluster is highly unlikely to bind to protein families which share no known high-affinity records with itself. Therefore, for any given compound with recorded interactions with fewer than six protein families, we used the protein families with no recorded interactions as the source of PN targets. PN targets were sampled to achieve compound-level balance. The rectified half sigmoid function was again used to determine the up-sampling negative-to-positive ratio based on the original compound-level negative-to-positive ratio.

### Data Splitting for Mimic Real Word Drug Discovery Scenarios

We categorize targets from records prior to 2015–2018 as the training data set, and those after the threshold year as the validation data set. The exact cutoff years may vary depending on the number of records for each protein family across different years. Four distinct validation data sets were constructed:

- Seen target and seen compound cluster for drug repurposing evaluation: this validation set includes targets and compounds previously examined in training data sets. These records provide experimental evidence for novel compound–protein pairs where the target and compound cluster were each previously studied in different contexts. Strong predictive performance on this data set suggests that the CPI model is effective for drug repurposing.
- Seen target and unseen compound cluster for novel lead compound discovery: this validation set includes records featuring seen targets but with compounds that differ significantly from those in training data sets. While drug repurposing aids in identifying compounds with known drug-likeness, this allows us to evaluate the model’s performance in the task of screening novel compounds.
- Unseen target and seen/unseen compound cluster for generalization: similarly, we considered scenarios involving the screening of seen compounds for unseen targets, and even more challenging, screening unseen compounds for unseen targets. These two data sets aid in evaluation by simulating situations where novel druggable targets are identified. The former can also be recognized as drug repurposing, while the later indicates a more challenge chemical space explored during virtual screening.

### Sequence-Based Compound–Protein Interaction Deep Learning Models

The concept of sequence-to-drug has recently emerged as a novel approach to virtual screening. Much like AlphaFold 2, which bypasses secondary structure prediction and directly forecasts 3D structures through end-to-end differentiable learning, sequence-to-drug models seek to identify lead compounds directly from protein sequences without intermediary steps, employing the same end-to-end differentiable learning approach. Considering various molecular encoders, protein encoders, and interaction modules, 10 representative methods have been selected for benchmark evaluation in this study. The key features of these methods are outlined below

- Protein sequence encoder: DeepDTA, DeepConv-DTI, GraphDTA, HyperAttentionDTI, and DrugBAN utilize convolutional layers to generate learned embeddings of amino acid sequences. MGraphDTA employs a multiscale network to learn across local and global structures of the compounds. BACPI introduce a context window to split the protein sequences into overlapping subsequences of amino acids, while MolTrans segments protein sequences into substructural patterns to learn at that scale. AI-Bind obtains feature vector for each protein sequence using pretrained method ProtVec. PMF-CPI relies on the protein language model TAPE-BERT.
- Compound representation: DeepDTA and HyperAttentionDTI process compounds as SMILES strings and utilize convolutional layers or LSTM to extract features. DeepConv-DTI employs calculated fingerprints as inputs for convolutional layers. GraphDTA, MGraphDTA, BACPI, PMF-CPI, and DrugBAN each develop their unique graph neural networks for compound representation. MolTrans segments compounds into substructural patterns similar to how it handles proteins. AI-Bind utilizes Mol2Vec that pretrained on SMILES strings to obtain compound representations.
- Interaction module: DeepDTA, DeepConv-DTI, GraphDTA, MGraphDTA, and AI-Bind concatenate the representations of proteins and compounds for final prediction. MolTrans adopts the dot product as an aggregation strategy for the two representations, while Kronecker product is considered in PMF-CPI. HyperAttentionDTI introduces a novel attention block that mimics intermolecular and channel-wise interdependencies. BACPI develops a bidirectional attention neural network model to integrate the representations. DrugBAN utilizes a bilinear attention map to learn interactions between residues and atoms.

In addition, KPGT^36^ is selected as the representative compound pretraining methods for ablation study. ProtVec,^27^ TAPE-BERT,^28^ ESM2-8M,^29^ ESM2-15B,^29^ and Ankh Large^30^ are also chosen as protein encoders of ablation study to study the impact of protein language models with varying parameter sizes.

### Downstream Evaluation

- Evaluating attention interpretation using PDBbind: we downloaded the general set of protein–ligand complex structures from PDBbind2020, comprising 19,443 entries. Following the same preprocessing protocol as MONN,^37^ we cleaned the data set and removed entries overlapping with the training data set. This yielded 9868 complex structures with annotated binding pockets for attention mechanism interpretation.
- Compound library for virtual screening: for virtual screening, we employed the Enamine Discovery Diversity Set (DDS-10), a high-quality library comprising 10,240 diverse compounds (covering 7253 distinct clusters).

## Supplementary Material



## Data Availability

The raw data used as benchmark are BindingDB available at http://www.bindingdb.org, ChEMBL database is available at https://www.ebi.ac.uk/chembl/, DrugBank data set is available at https://go.drugbank.com/, PDBbind 2020 avaliable at http://www.pdbbind.org.cn and Enamine available at https://enamine.net/compound-libraries/diversity-libraries. The CDPN data set is included in the GitHub repository at https://github.com/libokj/CDPN-VS. The CDPN data set generation and all models codes are available at https://github.com/libokj/CDPN-VS. DeepSEQreen is freely available at: https://www.ciddr-lab.ac.cn/deepseqreen/.

## Notes

Performance comparison between AI-Bind Network-Derived Negative Data set (NDN) and CDPN for model training (Figure S1); performance of Mol2Vec-ESM2-8M-MLP under various fine-tuning strategies for ESM2-8 M (Figure S2); comparison of different neural network architectures for processing features derived from TAPE-BERT (Figure S3); CPA and activity-cliff prediction performance across different models (Figure S4); performance evaluation of DeepConv-DTI on CPA prediction tasks with and without CDPN using four evaluation metrics (Figure S5); statistics from K-means clustering of PubChem compounds using varying K values (Figure S6); Venn diagram illustrating compound clusters experimentally tested in bioaffinity assays (Figure S7); negative prevalence before and after applying the rectified half-sigmoid function (Figure S8); summarized common CPI data sets (Table S1); ratio of positive records in different data sets (Table S2); target-level data distribution before and after CDPN (Table S3); compound-cluster data distribution before and after CDPN (Table S4); systematic evaluation of the interpretability of attention modules on PDBbind at the interaction-site level (ground truth calculated by PLIP) (Table S5); Onapristone rank for ADGRD1 under different models (Table S6); Nirogacestat rank for PSEN2 under different models (Table S7); and impact of different g and m parameter values in the sampling-ratio scale function on balancing negative prevalence (Table S8) (PDF)
