# Supplementary material for: Benchmarking Sequence-Based Compound–Protein Interaction Prediction through Constructing a Debiased Data Set CDPN
Source: J Chem Inf Model. 2025 Nov 20;65(23):12737–51. doi: 10.1021/acs.jcim.5c02040 (PMC12690583; doi:10.1021/acs.jcim.5c02040)
Supplement: Supplementary file 1 [file ci5c02040_si_001.pdf]

Benchmarking sequence-based compound-protein interaction prediction through constructing a debiased dataset

Yang Hao[1,2,3,#], Bo Li[2,3,#], Daiyun Huang[2,5,*], Lei Fu[2], Zhiwei Cao[4], and Xin Liu[2,*]

[1]Department of Hepatobiliary Surgery, Haikou Affiliated Hospital of Central South University Xiangya School of Medicine, Haikou 570208, China
[2]Wisdom Lake Academy of Pharmacy, Xi'an Jiaotong-Liverpool University, Xi'an Jiaotong-Liverpool University, Suzhou 215123, PR China
[3]Institute of Systems, Molecular and Integrative Biology, University of Liverpool, Liverpool L69 7ZX, UK
[4]School of Life Sciences, Fudan University, Shanghai 200092, China

[#]These authors contribute equally to this work.
*To whom correspondence should be addressed. Tel: +86 512 8188 9033;
Email: xin.liu@xjtlu.edu.cn
Correspondence may also be addressed to Daiyun Huang. Tel: +86 512 8188 3213;
Email: daiyun.huang02@xjtlu.edu.cn

Table S1 Summarized Common CPI Datasets.

Dataset	# of Interaction	# of Protein	# of Compound	# of Compound Cluster	# of Compound Cluster Covering 90% Compounds
Davis	30,056	442	68	64	58
BindingDB (v202310, cleaned)	1,549,652	6,548	699,875	48,893	18,943
KIBA	118,254	229	2,111	1,052	589
DrugBank (v5.1.5)	12,674	3,348	5,877	3,436	2,094
PDBbind (v2020)	15,201	852	11,846	6,894	5,374
Human (incl. screened negative samples)	6,728	2,001	2,726	1,542	870
C. elegans (incl. screened negative samples)	7,786	1,876	1,767	1,092	491
ChEMBL (v33, cleaned)	813,651	3,493	364,753	38,462	15,709
DUD-E	823,142	102	735,394	56,484	28,231

*Numbers in the table only show total data after data processing.

Table S2 Ratio of Positive Records in Different Datasets.

Dataset	# of Compound Cluster Covering 90% Compounds	# of Positive Compound Clusters	# Over-positive % (POS:NEG > 5:1)	# Over-negative % (NEG:POS > 5:1)
Davis	37	64	0	57.52
BindingDB (v202310, cleaned)	13,579	37,634	18.07	36.76
KIBA	570	1,030	49.34	0
DrugBank (v5.1.5)	2,094	3,436	100	0
PDBbind (v2020)	3,950	4,904	38.38	39.11
Human (incl. screened negative samples)	629	965	29.49	58.57
C. elegans (incl. screened negative samples)	394	766	24.95	57.30
ChEMBL (v33)	13,271	30,639	16.95	34.64
DUD-E	4,971	8,305	46.08	52.94

Table S3 Target Data Distribution Before and After CDPN.

	Non-CDPN	CDPN
N clusters	40897	40446
Cluster size mean	23.75	18.34
Cluster size median	6	9
N targets	3911	3879
Median cluster size	6	9
Mean cluster size	23.75	18.34
Max cluster size	2587	1227
% clusters > 50 size	10.37	7.29
#Unique cluster-target pairs	278330	367156
Mean records cluster-target per pair	3.49	2.02
Max records cluster-target per pair	1366	82

Table S4 Compound Cluster Data Distribution Before and After CDPN.

	#Clusters	# 90% Positive Clusters	# Positive Clusters	# Over Positive (POS:NEG > 5:1)	Over Positive %	#Over Negative (NEG:POS > 5:1)	Over Negative %
Non-CDPN	40897	8934	33180	10719	26.21	8860	21.66
CDPN	40446	0	32947	0	0	9268	22.91

Table S5 Systematically Evaluation of Interpretability of Attention Modules on PDBbind at Interaction Site Level (ground truth calculated by PLIP).

Compound Encoder	Protein Encoder	Decoder	Mean Coverage	Mean IoU
KPGT	Ankh Large	BIDAT	0.30	0.02
KPGT	Ankh Large	BAN	0.23	0.02
GraphSAGE	TAPE-BERT	BIDAT	0.13	0.02
GraphSAGE	TAPE-BERT	BAN	0.32	0.02

Table S6 Onapristone Rank for ADGRD1 under Different Models.

Model	Rank	Predicted Probability
DrugBAN	3787	0.236
GraphDTA	4893	0.129
MGraphDTA	4700	0.449
KPGT_Ankh_BAN	852	0.652
KPGT_Ankh_BIDAT	**92**	0.777
KPGT_Ankh_MLP	**27**	0.859
Mol2vec_ESM2_MLP	1096	0.473

Table S7 Nirogacestat Rank for PSEN2 under Different Models.

Model	Rank	Predicted Probability
DrugBAN	**13**	0.844
GraphDTA	111	0.859
MGraphDTA	653	0.746
KPGT_Ankh_BAN	699	0.711
KPGT_Ankh_BIDAT	**25**	0.844
KPGT_Ankh_MLP	**56**	0.855
Mol2vec_ESM2_MLP	200	0.605

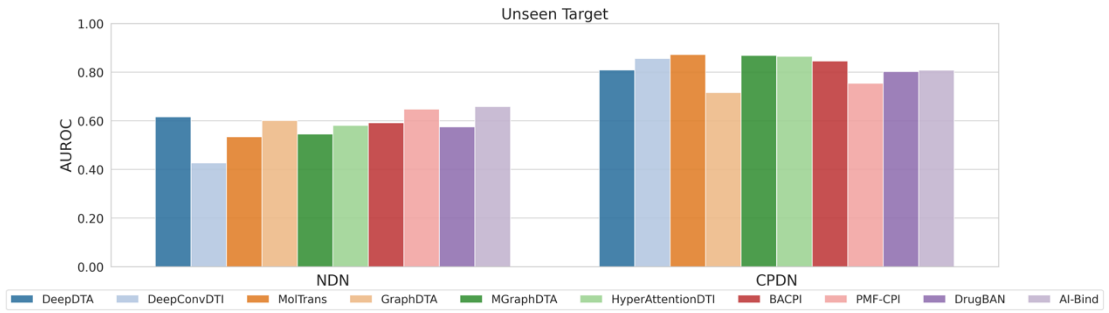

Figure S1. Performance comparison between AI-Bind Network Derived Negative Dataset (NDN) and CDPN for model training. Unseen target: protein target not covered in training dataset. The AI-Bind training data were obtained from its supplementary data. The test datasets are same, only the training and validation datasets are different between NDN and CDPN.

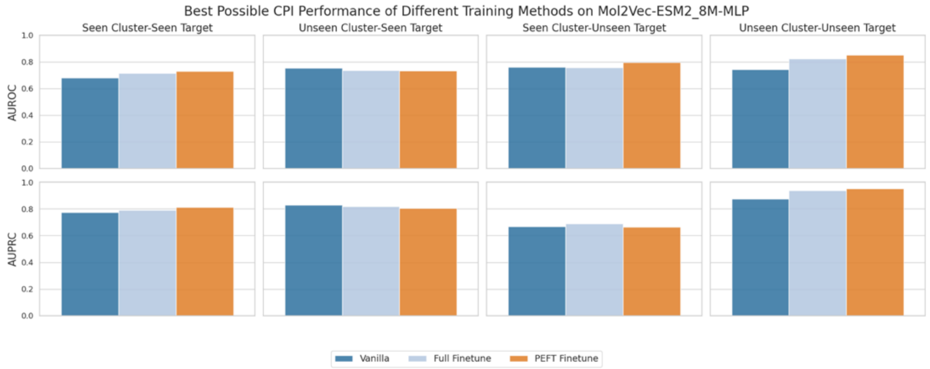

Figure S2. The performance of Mol2Vec-ESM2-8M-MLP under different fine-tuning strategies for ESM2-8M.

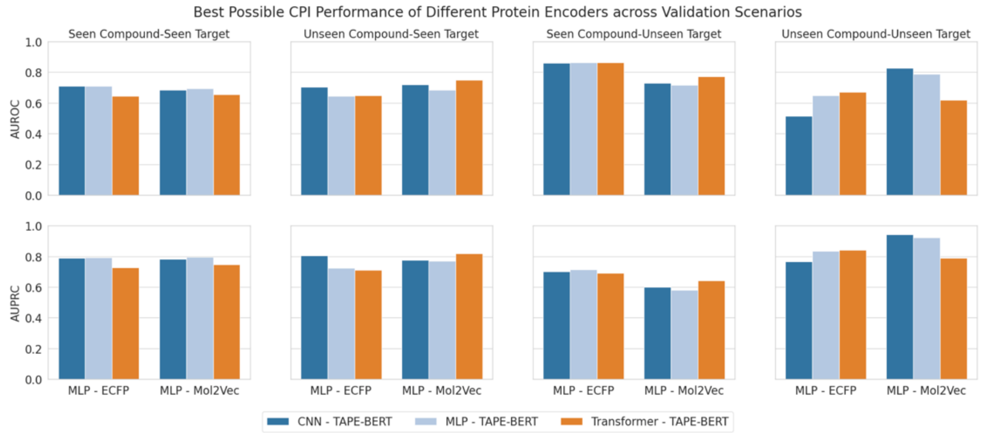

Figure S3. Comparison of different neural network to process the features derived from TAPE-BERT.

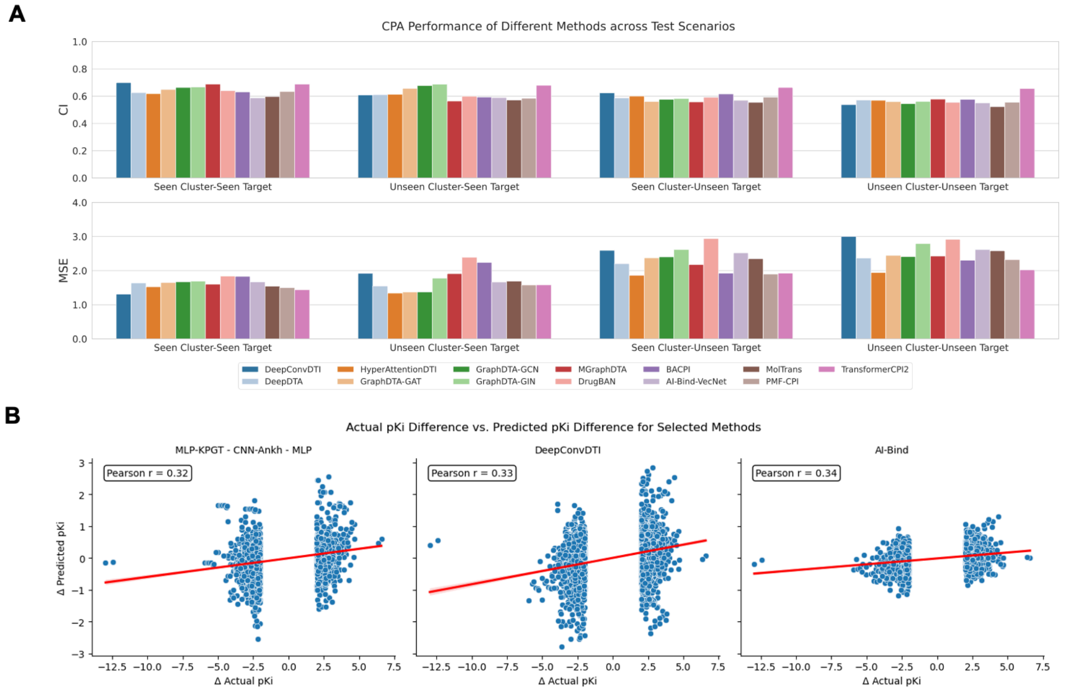

Figure S4. CPA and activity cliff performance across different models. A MSE and CI for 13 existing models on four scenarios. **B** Pearson's correlation coefficient between actual and predicted Ki difference for activity cliff pairs. The dataset was curated from ChEMBL, BindingDB, and PDBbind using a procedure similar to that of CDPN. Both IC50 and Ki data were utilized for benchmarking, and the observed trends were consistent across these datasets. For the activity cliff analysis, pairs were retrieved following previous studies, with only pKi differences above 2 being considered, corresponding to approximately a 100-fold difference in affinity. To balance the affinity prediction, putative negatives derived from CDPN were employed, with normally distributed low affinities assigned randomly. Additionally, a Gaussian-penalized mean squared error (MSE) loss was used. However, no significant performance improvement was observed, highlighting the significant challenges in balancing CPA tasks.

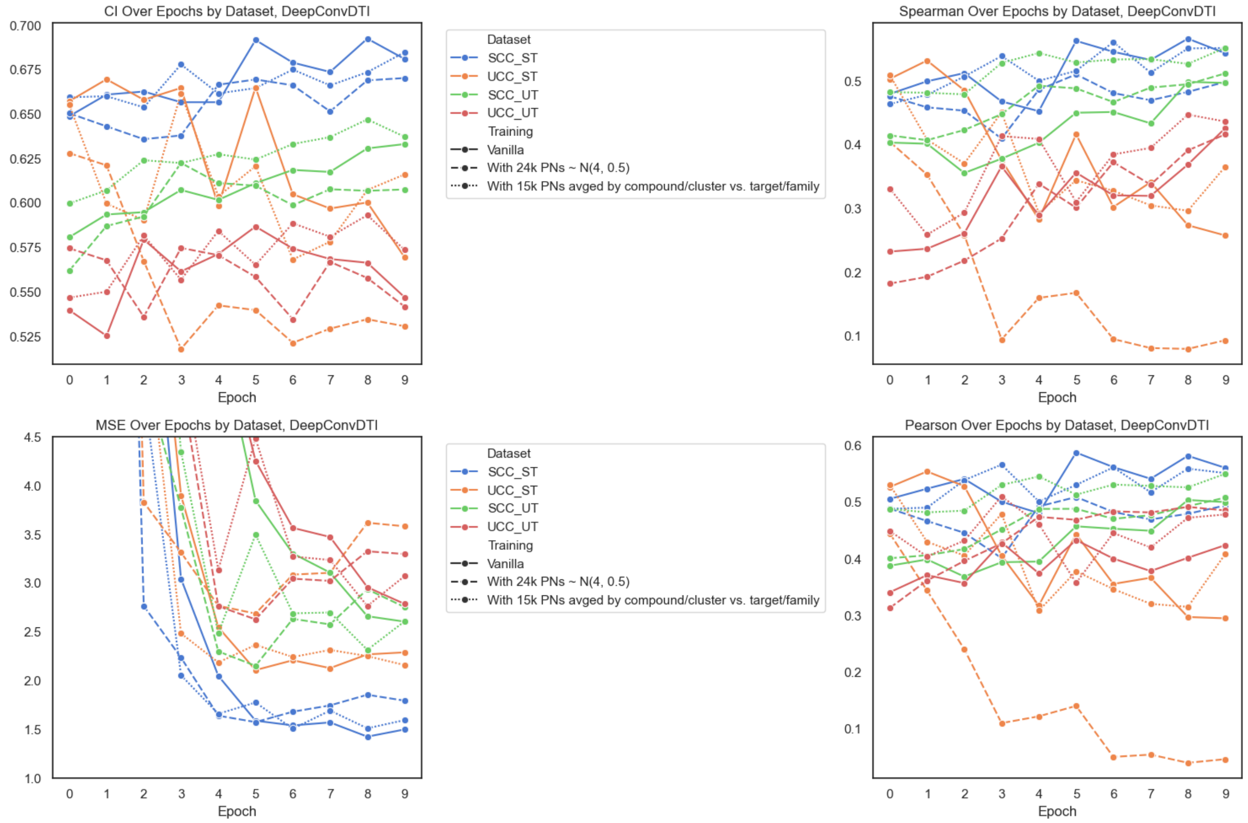

Figure S5. Performance evaluation of DeepConvDTI on CPA prediction tasks with and without CDPN, assessed using four evaluation metrics.

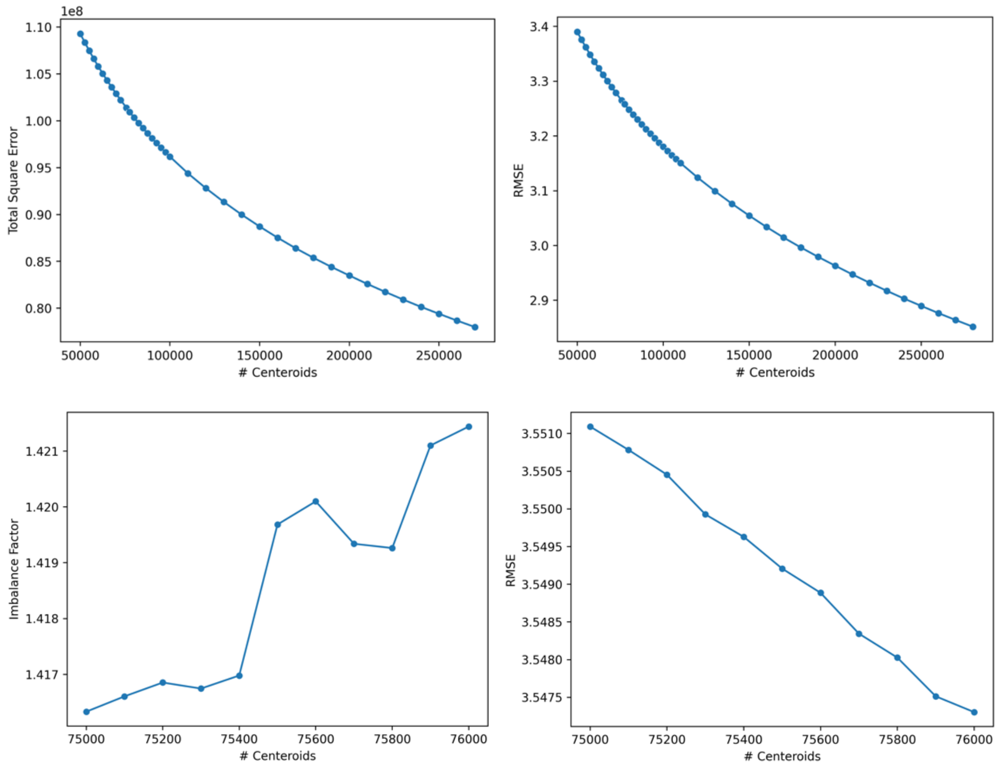

Figure S6. Statistics from K-means clustering of PubChem compounds using varying values of K.

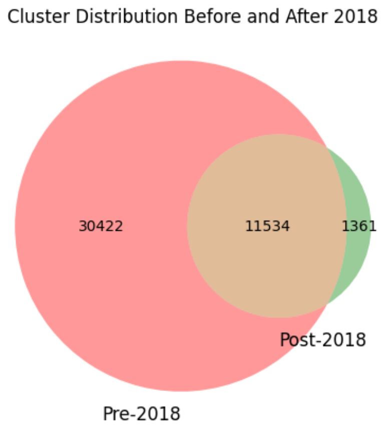

Figure S7. Venn diagram illustrating the compound clusters experimentally tested in bioaffinity assays.

Table S8 Impact of Different *g* and *m* Parameter Values in the Sampling Ratio Scale Function on Balancing Negative Prevalence.

g	m	Number of Putative Negative Pairs Added	Overall Negative Prevalence	Target-Wise Negative Prevalence (Mean ± Std)	Compound-Wise Negative Prevalence (Mean ± Std)
	30.0	215,262	0.619	0.617 ± 0.236	0.660 ± 0.256
2.0	40.0	254,844	0.638	0.633 ± 0.222	0.678 ± 0.239
	50.0	268,745	0.644	0.640 ± 0.217	0.680 ± 0.238
	30.0	191,833	0.607	0.602 ± 0.248	0.655 ± 0.257
3.0	40.0	205,689	0.614	0.612 ± 0.239	0.659 ± 0.256
	50.0	248,139	0.635	0.630 ± 0.224	0.677 ± 0.240
	30.0	165,977	0.592	0.588 ± 0.261	0.644 ± 0.267
5.0	40.0	181,368	0.601	0.601 ± 0.249	0.651 ± 0.261
	50.0	230,174	0.626	0.622 ± 0.232	0.674 ± 0.240

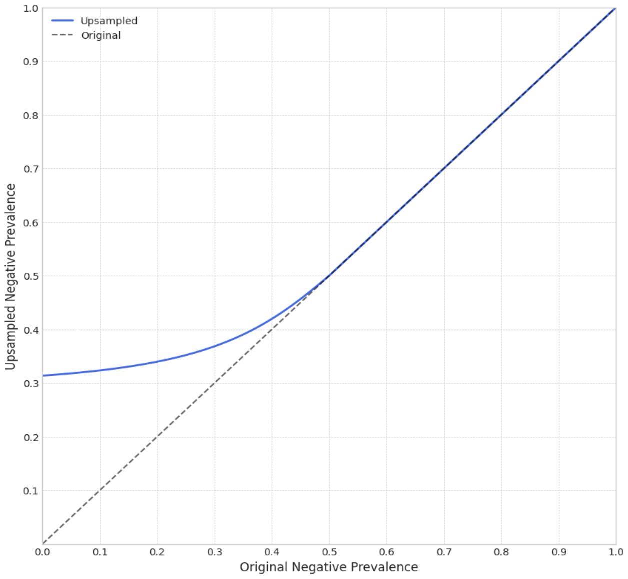

Figure S8. The negative prevalence before and after applying rectified half-sigmoid function.
